# Trends of global health literacy research (1995–2020): Analysis of mapping knowledge domains based on citation data mining

**DOI:** 10.1371/journal.pone.0254988

**Published:** 2021-08-09

**Authors:** Shaojie Qi, Fengrui Hua, Shengyuan Xu, Zheng Zhou, Feng Liu

**Affiliations:** 1 Research Institute of Social Development, Southwestern University of Finance and Economics, Chengdu, China; 2 Graduate School of Information Sciences, Tohoku University, Sendai, Japan; 3 School of Foreign Language, Huaiyin Normal University, Huai’an, China; College of Medicine and Sagore Dutta Hospital, INDIA

## Abstract

**Background:**

During uncertainties associated with the COVID-19 pandemic, effectively improving people’s health literacy is more important than ever. Drawing knowledge maps of health literacy research through data mining and visualized measurement technology helps systematically present the research status and development trends in global academic circles.

**Methods:**

This paper uses CiteSpace to carry out a metric analysis of 9,492 health literacy papers included in Web of Science through mapping knowledge domains. First, based on the production theory of scientific knowledge and the data mining of citations, the main bodies (country, institution and author) that produce health literacy knowledge as well as their mutual cooperation (collaboration network) are both clarified. Additionally, based on the quantitative framework of cocitation analysis, this paper introduces the interdisciplinary features, development trends and hot topics of the field. Finally, by using burst detection technology in the literature, it further reveals the research frontiers of health literacy.

**Results:**

The results of the BC measures of the global health literacy research collaboration network show that the United States, Australia and the United Kingdom are the major forces in the current international collaboration network on health literacy. There are still relatively very few transnational collaborations between Eastern and Western research institutions. Collaborations in public environmental occupational health, health care science services, nursing and health policy services have been active in the past five years. Research topics in health literacy research evolve over time, mental health has been the most active research field in recent years.

**Conclusions:**

A systematic approach is needed to address the challenges of health literacy, and the network framework of cooperation on health literacy at regional, national and global levels should be strengthened to further promote the application of health literacy research. In the future, we anticipate that this research field will expand in two directions, namely, mental health literacy and eHealth literacy, both of which are closely linked to social development and issues. The results of this study provide references for future applied research in health literacy.

## 1. Introduction

Health literacy is a concept that is constantly being developed. In recent years, governments, health professionals and researchers have paid increasing attention to it, and at the 9th Global Conference on Health Promotion in 2005, it was listed as the core topic [1]. Many countries have prepared or started to establish health literacy monitoring and evaluation systems, hoping to enhance the overall health of the population by improving people’s health literacy [2], and countries such as the United States, Canada, the United Kingdom, Australia and China have even made health literacy a national strategy. The WHO (2013) [3] defines this term as “the personal characteristics and social resources needed for individuals and communities to access, understand, appraise and use information and services to make decisions about health”. It is believed that health literacy can play an important role in encouraging people and communities to participate in health care and build their resilience, improve their health and well-being, address health inequities, etc. The WHO has pointed out that health literacy is generally low in both developed and developing countries [1]. Evidence supports that increasing age, low educational attainment, disadvantaged socioeconomic status and poor reading level are the main barriers to health literacy. In addition to other socioeconomic issues, the literature shows that people with low levels of health literacy around the world also have a misunderstanding of health information in English [4]. In contrast to developed areas that are actively exploring interventions to improve people’s health literacy, the relevant research in some developing countries is still in its infancy. Studies have shown that low-income populations primarily have low reading skills, leading to their low health literacy [4, 5], especially in countries that are densely populated and ethnically and culturally diversified but heavily engaged in human development, economic stability and primary health care. At the same time, these countries are faced with great challenges in terms of providing health services to disadvantaged groups with low literacy rates and low socioeconomic status [6]. Thus, improving health literacy is an urgent need to facilitate and achieve the health-related United Nations Millennium Development Goals.

Generally, people with good health literacy can manage their health more effectively than those without or with poor health literacy [7]. The 2020 global pandemic of COVID-19 shows that low health literacy is a public health problem that has long been underestimated worldwide [8–10]. For example, nearly half of European adults mentioned that they had inadequate health literacy for taking care of their own health issues as well as those of others [11]. However, substantial evidence has proven that health literacy is an important tool for the prevention of noncommunicable diseases (NCDs), and sustainable long-term measures need to be implemented as early as possible [12]. In particular, in the current global epidemic, which is full of uncertainties, people need to quickly change their health cognition and behaviors to reduce the risk of infection and transmission of the disease. Therefore, how to improve the health literacy level of global citizens is of unprecedented importance.

Research on health literacy, as an emerging field, has grown rapidly over the past 30 years. The connotations have been enriched and specified, and the theories and methods involved have become much more extensive and complex. At the same time, research is expanding in the context of interdisciplinary approaches, with new theories and methods constantly being created, so determining field divisions might not be easy. At present, many scholars have reviewed this research topic in terms of basic concept and framework [7], measurement [13], evaluation methods [14] and impact mechanism [15]. To some degree, these studies have provided theoretical and methodological support for better understanding the involved interdisciplinary approaches to health literacy research, but the analytical perspectives have been relatively singular, and there is a lack of comprehensive and systematic studies on the development track of the discipline. Some questions still need to be answered: 1. Which main bodies (country, institution and author) promote research on health literacy, and what kinds of collaboration exists among them? 2. In the development process of health literacy research, which publications have acted as pioneers or played key roles? 3. What are the main topics in the research field, and how are the different research topics related to each other? Traditional review articles cannot comprehensively summarize and quantitatively analyze how knowledge in a certain field has developed across a large amount of literature, and their comments are usually qualitative in nature and prone to subjectivity.

As an important branch of science, bibliometrics has evolved into a relatively mature theoretical and methodological system after decades of development [16]. Based on scientific papers and citation data, it shows the features of attractiveness and objectiveness, explores the developmental characteristics and evolutionary rules of contemporary scientific research from different dimensions and perspectives, and provides an in-depth quantitative understanding of the relationships among scientists, research institutions and discoveries. Additionally, it plays a unique role in quantifying knowledge production and laws of scientific development [17, 18]. In addition, the scientific citation database, which records all the progress that has been made, is constantly being enriched and improved, providing an objective data basis for the study of science [19, 20]. Therefore, drawing knowledge maps of health literacy research through data mining and visualized measurement technology helps systematically present the research status and development trends in global academic circles and supplements the existing bibliometric research in this field.

On the basis of the previous unsolved questions, this study draws knowledge maps of global health literacy research by applying the existing bibliometric theory and data mining technology to outline the overall knowledge structure and development trends in the field and to better understand its research topics and evolution over time. The specific objectives of this study are as follows: 1. Construct the collaboration networks of health literacy research to understand the characteristics of research subjects, including the composition and collaboration modes of authors, the heterogeneity and collaboration modes of authors’ institutions, and the distribution and collaboration modes of authors’ countries. 2. Generate the cocitation networks of health literacy research to analyze the characteristics of key articles and citation structure. 3. Create the timeline view of health literacy research. Based on the different topics and changing trends of health literacy research, this paper reveals the research frontiers and future propositions of the field.

## 2. Methodology

### 2.1. Data source and processing

The scientific nature of mapping knowledge domains is rooted in their databases; that is, how to accurately and comprehensively retrieve all the documents on a subject is the key to data collection. Therefore, the data sources used for CiteSpace should be authoritative and massive at the same time. The Web of Science (WoS) Core Collection is selected in this paper since it has been trusted and selected as a global citation database over time [21, 22]. First, WoS has been widely recognized as a great citation database for bibliometric research and the largest comprehensive scholarly information resource covering peer-reviewed journals with high impact factors [21, 23–25]; the core WoS database consists of over 21,419 types of journals, books and conference proceedings with over 79 million records (WoS, October 2020). Chen (2016) [26] advocated that when people use scientometric analysis tools such as CiteSpace, the use of WoS as a data source can effectively prevent data loss and speed up data conversion. Our concrete retrieval strategies and data processing are as follows. First, publications with the subject term “Health Literacy” were searched in WoS, and the search was further optimized by the following conditions: language = English; document type = article + review. The number of search results was 9,888 (downloaded on September 19, 2020). Therefore, the period of the citing articles in our study is from January 1, 1995, to September 19, 2020. During the deduplication process, we excluded duplicate publications and articles with missing key fields, such as abstracts, keywords and references, resulting in 9,429 valid records for inclusion. Second, 245,234 citation datasets for document cocitation analysis were established from the 9,429 papers and divided into citing articles and cited references. Citing articles, also known as source articles, are the main parts of the data records in WoS, including information such as author, institution, country, title, keywords, category and cited references. Cited references are usually documents of high quality, including the author, title, journal, year and other information, and can be cited many times by others. The parameters of the collaboration analysis and document cocitation analysis in CiteSpace were set as shown in Table 1.

**Table 1:** Parameters of collaboration network analysis.

Analysis Types	Time	Node Types	Years Per Slice	Selection Criteria	Nodes	Links
Co-author Collaboration	1995–2020	Author	1	Top N = 100	2343	5558
Co-institution Collaboration	1995–2020	Institution	1	Top N = 100	1275	7920
Co-country/region Collaboration	1995–2020	Country/region	1	Top N = 100	89	955
Interdisciplinary Collaboration	1995–2019	Category	5	Top N = 100	194	1482
Document Co-citation Analysis	1995–2020	Reference	1	Top N = 100	952	6099

### 2.2. Analysis

#### 2.2.1 Data mining and visualization

Data mining is a process of exploring the potential relationship between large amounts of disordered data and determining the information that may be ignored, hidden or unknown [27]. In recent years, data mining techniques have been developed rapidly in new research fields such as social network analysis, image data mining, and structural and temporal data analysis [28]. The visualization techniques transform the data mining results from abstract results into graphics or images with the help of visualization models and form rich and meaningful infographics and mapped knowledge domains, thus building a “bridge” between data mining and knowledge discovery [29, 30]. This paper uses the visualization software CiteSpace (CiteSpace 5.6.R4 Version) to carry out data mining and visual analysis of the scientific literature of health literacy to identify its knowledge bases, research hot spots and development trends on the basis of big data and provide references for future studies.

CiteSpace is a Java application for visualizing information in scientific literature, based mainly on cocitation analysis theory and pathfinder network scaling (PFNET). It facilitates a systematic review of a progressive knowledge domain with pivotal points and intellectual turning points as well as potential dynamic mechanisms and frontiers through a series of visualization maps [31]. Therefore, the software can effectively help readers better understand the research field in which they are engaged because it not only shows the overall situation of a certain area but also highlights specific important literature in the development process.

#### 2.2.2 Multilevel collaboration network analysis

Scientific collaboration refers to the research of scholars who create new scientific knowledge together, and their collaboration network represents the details of their research field. Specifically, the more frequently they collaborate, the greater depth their discipline develops [21]. In reality, scientific collaboration has multiple forms and manifestations, but this study focuses mainly on collaboration between different countries/regions, institutions or authors occurring in the same paper. Through CiteSpace, three levels of analysis of scientific collaboration networks are provided: macro co-country/region, meso coinstitution and micro coauthor. In addition, the dynamic structure of interdisciplinary collaboration in health literacy research over the past 25 years is drawn in this paper and can be used as a guide for new researchers as well as researchers seeking potential collaboration.

A collaborative network can be specifically analyzed through a visualized graph and the measurement of BC between collaborators. Betweenness centrality, which quantifies the significance of a node in a network, is used in CiteSpace to reveal the importance of a document contributor (country, institution, or author), as well as to mark the key point of a node in a purple circle. This method of calculating the importance of a node was proposed by Freeman in 1997 [32], the formula which is as follows:

$${BC}_{i}=\sum_{s\neq i\neq t}\frac{n_{st}^{i}}{g_{st}}$$

where ***g***_***st***_ is the number of shortest paths from node s to node t, among which $n_{st}^{i}$ is the number of shortest paths passing through node i. From the perspective of information transmission, the higher the BC is, the greater the influence of the node. If the node is removed, it will have a strong impact on network transmission.

#### 2.2.3. Multiperspective document cocitation analysis

Cocitation analysis is one of the most commonly used methods in scientometrics, first proposed in 1973 by Henry Small, an American information scientist [33]. It refers to pairs of papers that are cited together in source articles. The higher the frequency of cocitation is, the closer the relationship and the more similar the academic background of the two documents. In this paper, document cocitation analysis, which means the process of mining the cocitations in a document’s structural data clusters, is mainly applied [34]. Through this process, researchers are able to better understand and quantitatively reveal the structure, relationship and evolution of science as well as the research frontiers of a discipline [31]. Additionally, this method provides a basic clustering mechanism for cocitation networks so that researchers can use it to identify different research branches and hot topics [21, 35, 36]. Traditional cocitation analysis generally emphasizes citing articles, which are used as the source and basis of identifying terms for clusters. This paper uses CiteSpace’s cocitation analysis method from multiple perspectives to explore the citation networks and cluster structure of health literacy research by considering both cited references and citing articles.

**Fig 1:**
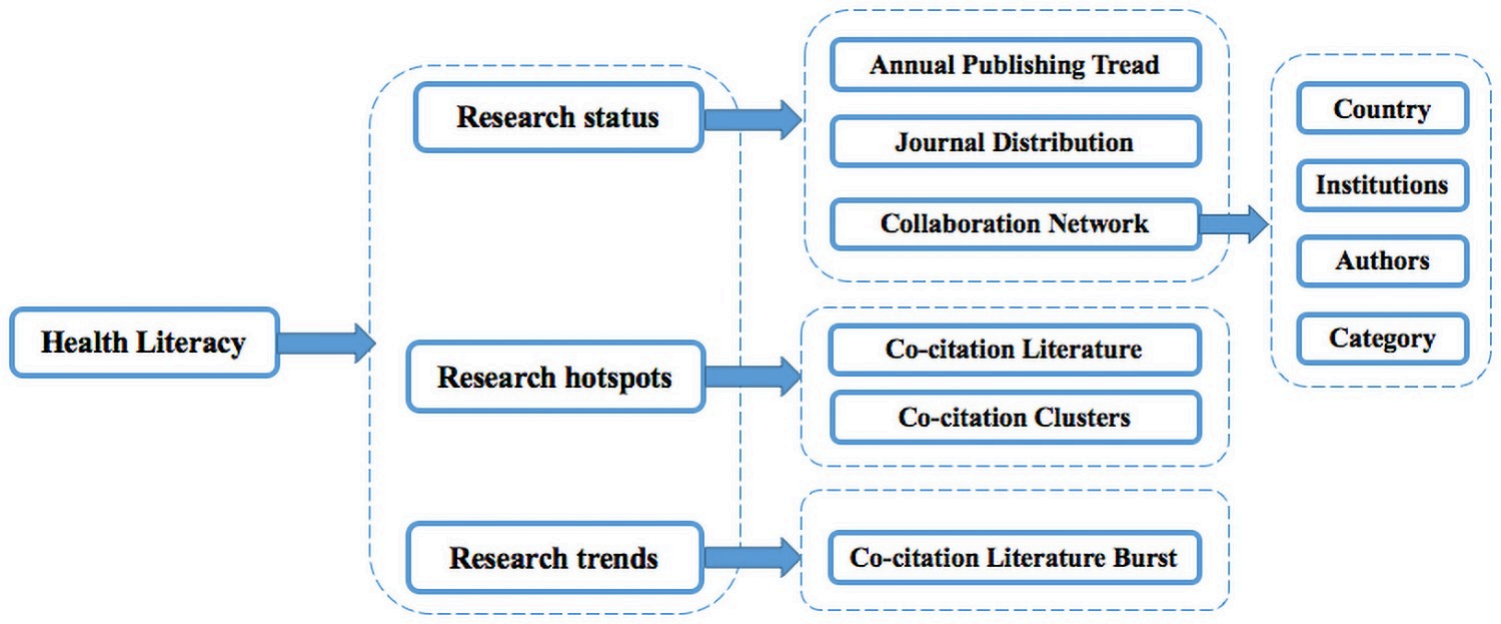
Research framework.

To realize the clustering networks, we use the log-likelihood ratio (LLR) provided by CiteSpace, which helps name the clusters by extracting similar items in titles, keywords or abstracts and calculating the similarity rates [37]. The LLR tends to reflect the uniqueness of a cluster and is more suitable for generating high-quality clusters with intraclass and interclass similarity [38]. Therefore, the LLR algorithm is mainly used in the clustering analysis section of our study. In addition, regarding the document cocitation analysis that reveals the relevant research frontiers, we adopt another key CiteSpace measure, i.e., burst detection. Burst detection can detect great changes in the amount of information being cited and regards the mutation of information as a means of measuring profound changes to determine the rise or fall of interest in certain cited references or a topic and to predict the frontier of a certain research field [21, 31, 39, 40]. In particular, the continuous bursts in recent years may lead to new research trends, and the specific analytical framework of this study is shown in Fig 1.

## 3. Results

### 3.1. Basic attributes of health literacy research

#### 3.1.1. Global publications

**Fig 2:**
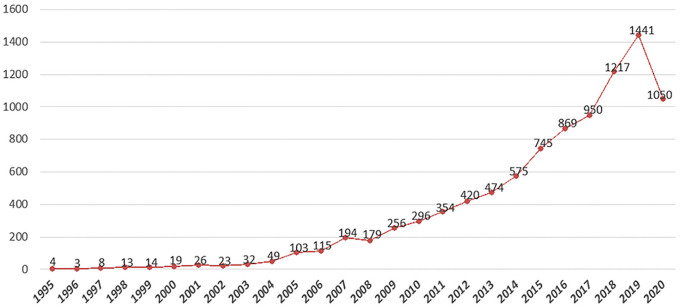
Health literacy trends from 1995 to 2020. In the past 26 years, a total of 9,492 articles on health literacy were published in the WoS Core Collection.

The change in paper number is an important indicator to measure the development trend of a research topic over a specific period of time. It can directly present the variation in research heat, which is of great significance in analyzing and predicting the future. Generally, if the number of publications in a research field increases over the years, it often means that this field has received continuous attention from scholars. Fig 2 shows the annual distribution of 9,429 papers on health literacy collected from the WoS database since 1995. Before 2000, the number was scarce, and the annual output was less than 20. Since 2005, the papers have increased significantly, with more than 100 published in a single year, showing an exponential growth trend. By 2019, the annual output of global health literacy papers had exceeded 9,000 and continued to show a high level of growth. Due to the time delay of the database, papers published in 2020 are not fully included and had decreased in number compared with the previous year.

#### 3.1.2. Main journals

As a typical interdisciplinary field, health literacy research covers a wide range of disciplines. Therefore, the distribution of publications in this field is relatively scattered. Papers are published in more than 1,600 journals, most of which are professional and comprehensive journals in the fields of medicine and public health. Table 2 lists the top 10 journals with the largest number of articles in the field, which cover approximately 20% of all health literacy papers. Among them, *Patient Education and Counseling* is the most important, with 351 papers published. It is followed by *Journal of General Internal Medicine*, a medical journal with 262 published articles. *Journal of Health Communication* is third, with only two fewer articles than the second-place journal. It is worth noting that health literacy, as a new research topic, has attracted much attention from open access journals such as *BMC Public Health*, *PLoS One* and *BMJ Open*, which in turn have to some extent accelerated the spread of research results in this field.

**Table 2:** Top 10 most prolific journals of health literacy.

Journal	Count	%	Impact factor(2019)
Patient Education and Counseling	351	3.72	3.408
Journal of General Internal Medicine	262	2.78	4.950
Journal of Health Communication	260	2.76	2.358
European Journal of Public Health	212	2.24	3.134
International Journal of Environmental Research and Public Health	182	1.93	3.127
BMC Public Health	167	1.77	3.182
PLoS One	143	1.52	3.226
Journal of Medical Internet Research	130	1.38	5.996
BMJ Open	113	1.20	2.992
BMC Health Services Research	112	1.19	2.564

### 3.2. Collaboration network analysis of health literacy research

Science can be considered an activity of exploring new knowledge with intelligence and social dimensions. Group interaction is the basic mechanism for the generation of scientific knowledge, while scientific collaboration is the most direct form [41]. In this paper, the size of nodes represents the frequency of research content published by countries/regions, institutions or authors, and the thickness of the connecting lines between nodes reflects the strength of the co-occurrence relation between the main bodies. In addition, based on Freeman’s algorithms for node influence, betweenness centrality (BC) is used to discover and measure the influence of the nodes [42, 43], and purple circles are used to mark these nodes. A node with high BC is usually a key hub linking two other nodes, also known as the turning point [31]. In addition, a color change in the diagram from a cold color (blue) to a warm color (yellow) represents the time sequence of collaboration.

#### 3.2.1. National influence and co-country/region collaboration network

From 1995 to 2020, 89 countries/regions participated in collaboration on health literacy research, and the top ten countries were selected according to the number of publications. There are differences in the numbers among countries. For example, as the largest producer, the United States published 4,939 papers, followed by Australia, with a total of 1,289.

**Fig 3:**
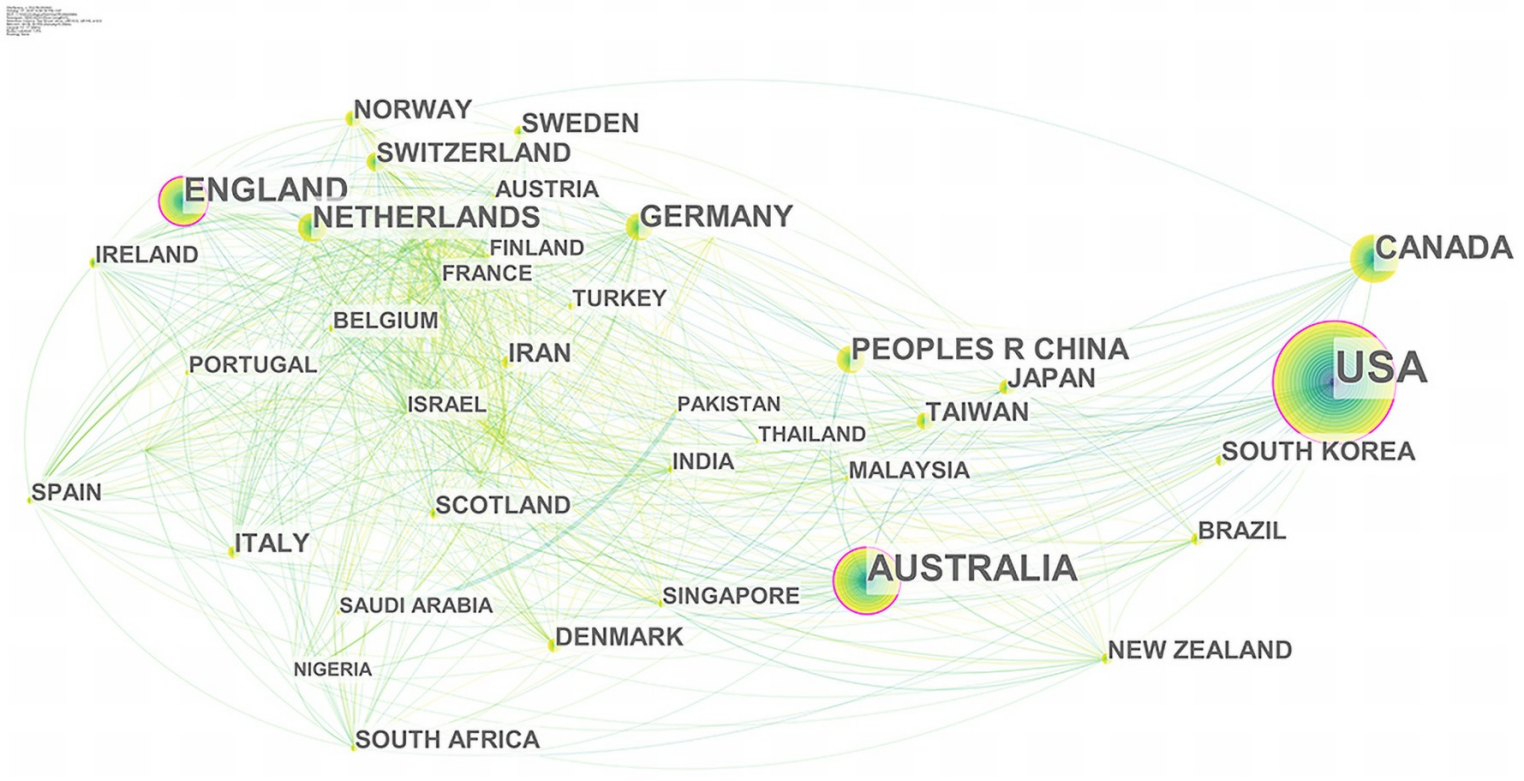
Country/region collaboration network of health literacy. Each node represents a country/region, and the size of the node indicates the number of publications of the country/region. Each edge indicates a collaborative relationship between countries/regions.

Fig 3 shows the co-country/region collaboration network of health literacy research. It shows that most countries have carried out cross-border cooperation in this field. The nodes of the United States, Australia and the United Kingdom have purple outer circles, indicating that the three have high BC (BC > 0.1) and occupy an important position in global research cooperation on health literacy, along with great impact.

#### 3.2.2. Core research institutions and the coinstitution collaboration network

From 1995 to 2020, 790 institutions participated in research collaboration on health literacy. Among them, Northwestern University in the United States published 306 papers, followed by the University Melbourne, Australia, with 272 publications (Table 3). There were 49 institutions with over 100 articles each. In general, the main forces in this field are concentrated in a few research institutions.

**Table 3:** Top 10 most productive countries, institutions and authors of health literacy.

Country	Count	BC	Institution	Count	BC	Author	Count	BC
USA	4939	0.13	Northwestern University	306	0.09	Michael S. Wolf	167	0.10
Australia	1289	0,09	the University of Melbourne	272	0.06	Anthon F. Jorm	72	0.01
England	623	0.09	the University of North Carolina System	237	0.11	Dean Schillinger	69	0.04
Canada	547	0.07	the University of Sydney	228	0.07	Sunil Kripalani	69	0.02
Netherlands	310	0.07	University of California, San Francisco	220	0.09	Richard H. Osborne	56	0.02
Peoples R China	302	0.03	Emory University	217	0.06	Michael K. Paascheorlow	55	0.04
Germany	299	0.05	Vanderbilt University	174	0.02	Terry C. Davis	47	0.01
Switzerland	165	0.03	Monash University	149	0.06	Russell L. Rothman	39	0.01
Sweden	157	0.03	The University of Michigan	143	0.06	Nicola J. Reavley	38	0.01
Japan	150	0.02	Harvard University	134	0.08	Laura M. Curtis	37	0.01

**Fig 4:**
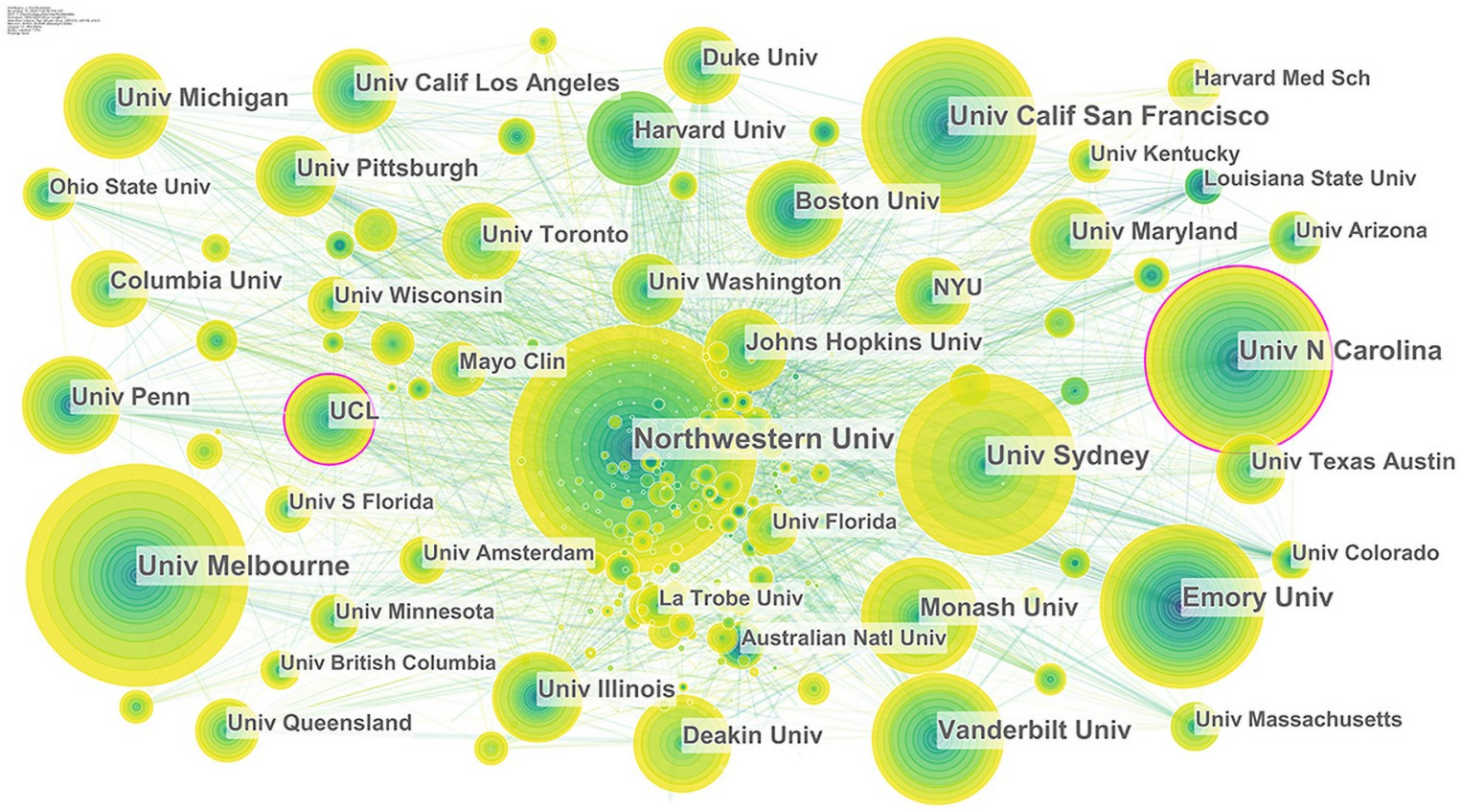
Institution collaboration network of health literacy. Each node represents an institution, and the size of the node indicates the number of publications of the institution. Each edge indicates a collaborative relationship between two institutions.

Cooperation among authors from different institutions can form a collaboration network reflecting their relationship. Health literacy research also possesses the characteristics of collaboration among different institutions (Fig 4). Specifically, the nodes of the University of North Carolina System and Northwestern University both have purple outer circles (BC > 0.1), indicating that the two are not only the main sources of publications on health literacy but also play an important role as bridges in the collaboration network.

#### 3.2.3. Core authors and the coauthor collaboration network

From 1995 to 2020, 1,807 authors participated in research on health literacy. According to the number of publications, Table 3 lists the top 10 authors in the field, among whom Michael S. Wolf ranks first, with 167 articles published. Another fruitful author is Anthon F. Jorm, with 72 publications.

**Fig 5:**
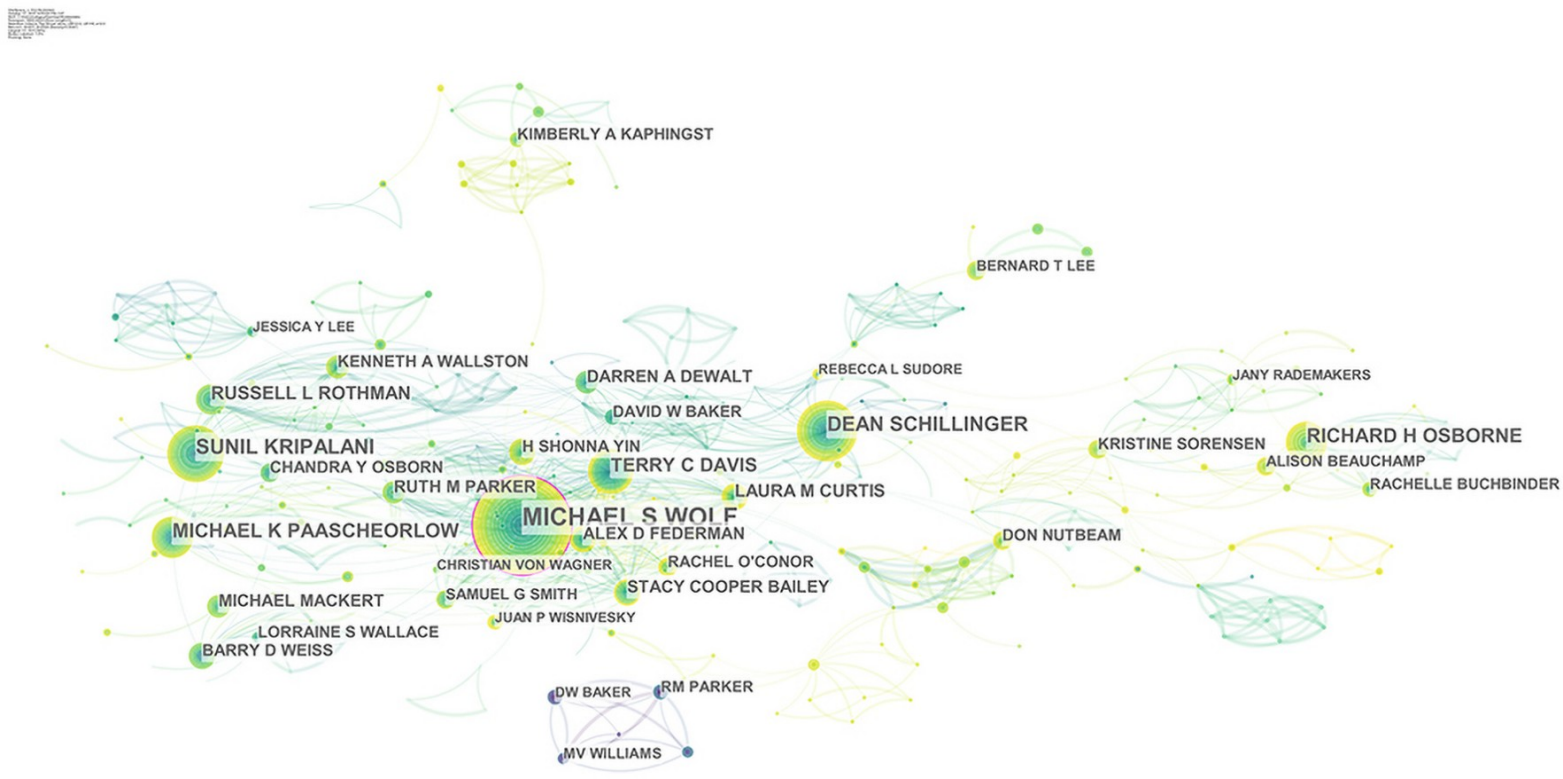
Author collaboration network of health literacy. Each node represents an author, and the size of the node indicates the number of publications of the author. Each edge indicates a collaborative relationship between two authors.

Authors and their social relations are the core elements as well as the key forces of a certain research field. Through the analysis of the coauthor collaboration network, we can determine which scholars cooperate closely in the field and further discuss the influence of team cooperation on their academic performance. From the coauthor collaboration network of health literacy research shown in Fig 5, we can infer that Michael S. Wolf, an American scholar with the highest BC, has greatly changed the network in his field and formed a strong team. In addition, the top three authors in health literacy are all highly correlated.

#### 3.2.4. Discipline evolution and interdisciplinary collaboration

**Fig 6:**
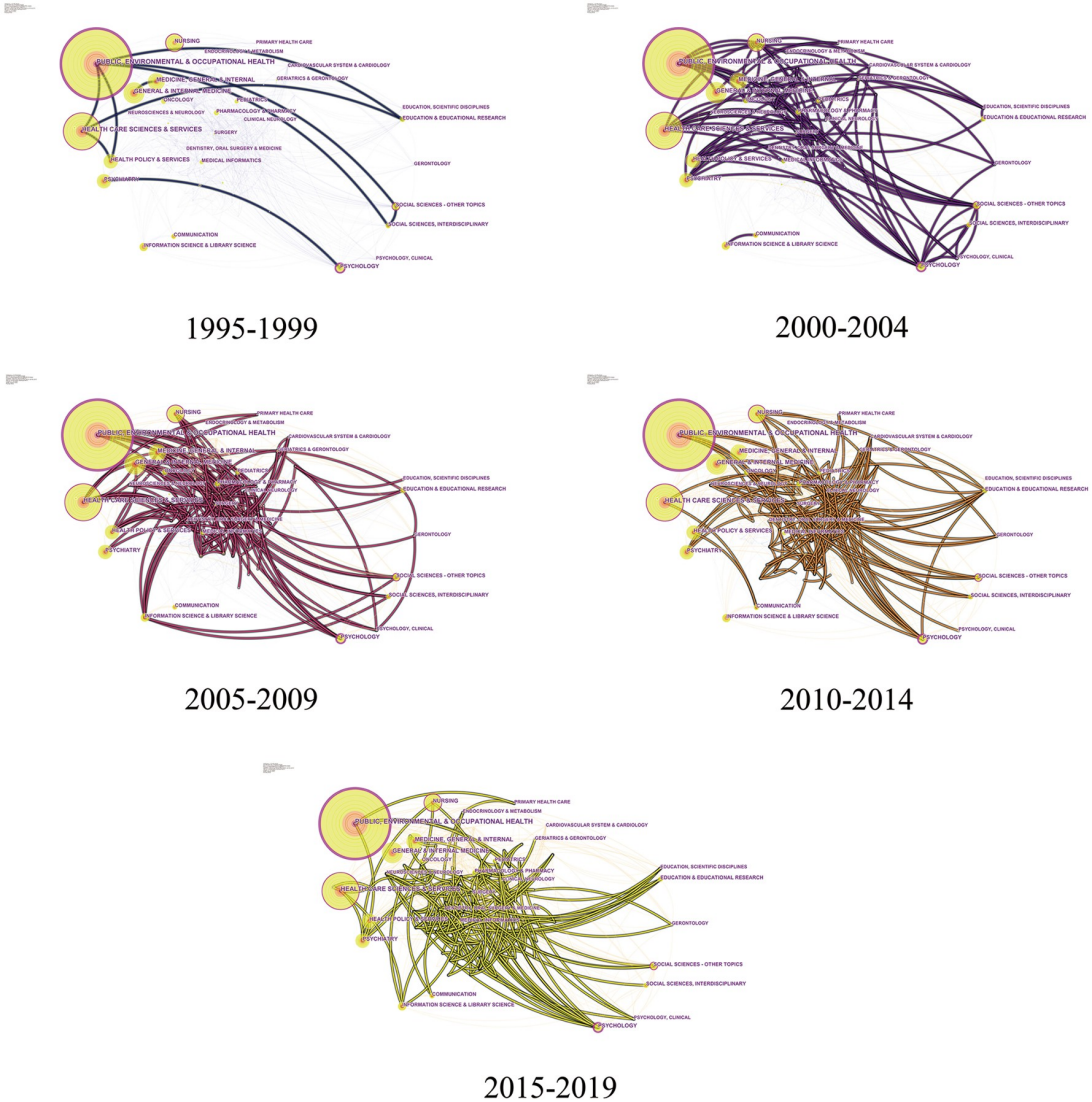
Category collaboration network of health literacy. Each node indicates a category, and the larger the node, the more papers were published. Each edge indicates a collaborative relationship between two categories.

To better understand the expansion and evolution of health literacy research, all papers in the field of health literacy since 1995 are divided into five stages, namely, 1995–1999, 2000–2004, 2005–2009, 2010–2014 and 2015–2019. Fig 6 shows the interdisciplinary collaboration of health literacy papers during these stages and reflects their tendency to move from medicine to other disciplines. From 1995 to 1999, research on health literacy was in its infancy, with a relatively small number of publications. In addition, it was distributed mainly in the areas of medicine and health care, indicating that its disciplinary attributes during this stage were highly concentrated. Since 2000, research on health literacy has matured with the rapid increase in the number of papers and expanded research fields. Interdisciplinary collaboration has been extended and deepened, with medicine, public health and health policy as the core disciplines and psychology, education and social science as the supplements. Additionally, the close similarity between 2010–2014 and 2015–2019 in the knowledge map means that global health literacy research has formed its own discipline cluster. In the past five years, computer science in information science and social work in applied social sciences have both turned their attention to health literacy.

### 3.3. Document cocitation analysis of health literacy

Citation is one of the core elements of academic work, through which scientific literature can either cite or be cited, forming an interconnected network of literature [44]. Citation analysis is a bibliometric method that takes citations as the research object and uses the methods of statistical analysis, network analysis and content analysis to examine the network pattern of scientific papers, authors, institutions, journals, etc. to reveal their quantitative characteristics and inherent laws and to study the dynamics of scientific documents. It is often used to identify and define research fields, discover and explore the knowledge community, analyze and predict research frontiers [34], etc. This study will identify the main research directions and core literature of health literacy through CiteSpace’s analytic function.

#### 3.3.1. Cocitation networks and key references

**Fig 7:**
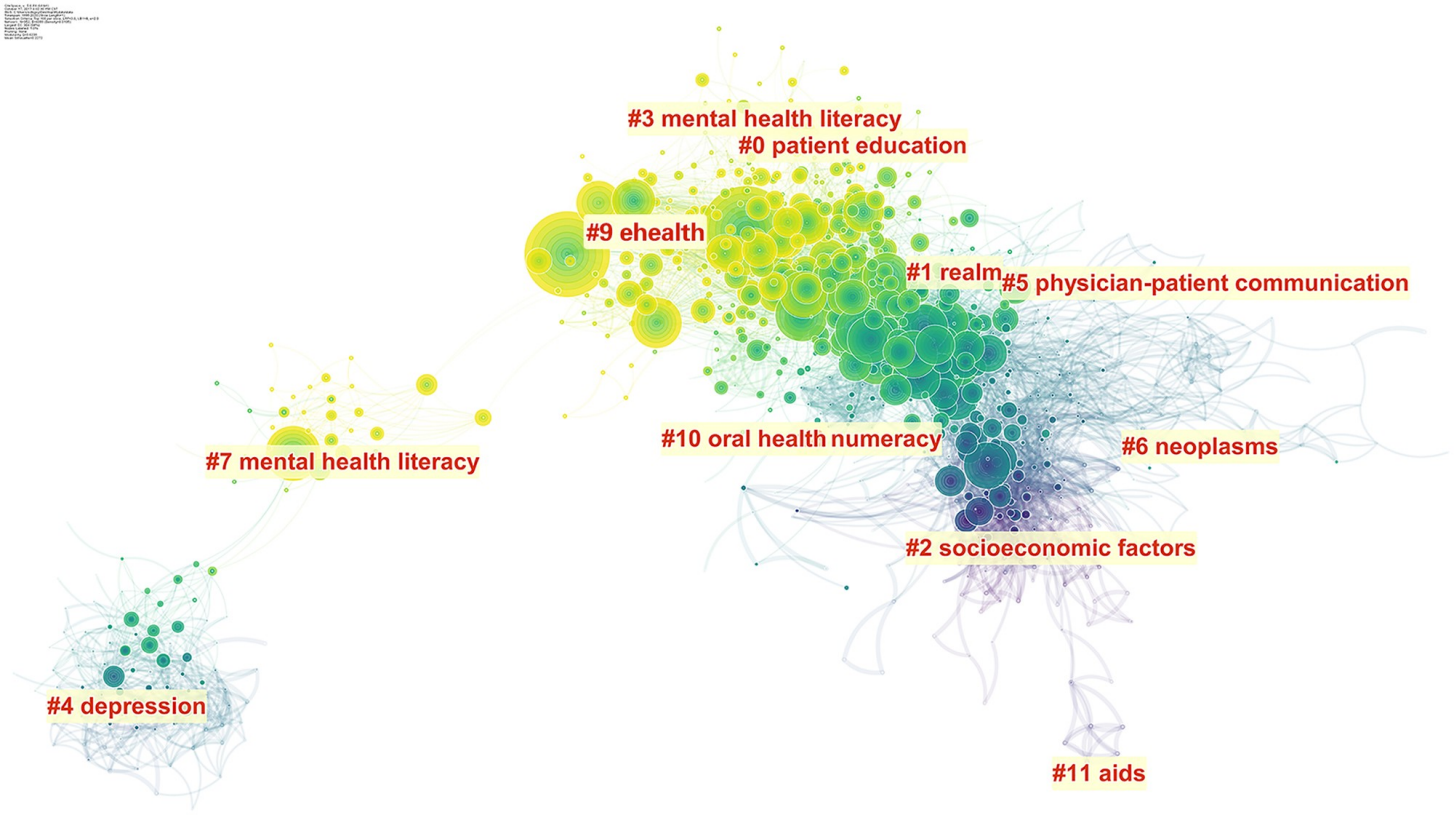
Category document cocitation clustering network of health literacy. A total of 12 clusters were generated in the graph. Each node represents one cited reference, and each edge indicates the cocitation relationship. The color represents the date of publication: yellow indicates literature that is newly published, and green and blue indicate literature published in earlier years.

To further understand the characteristics of the structure of the cocitation network of health literacy, CiteSpace is used to retrieve information regarding cited references represented by network nodes. The visualized network reveals the overall structure of health literacy research in a broader context (Fig 7). The network is composed of three differently colored regions: the right half of the network is basically blue, which indicates that the citation relationship of this part occurred mainly between 1995 and 2000; the middle part has blue and yellow links, most of which are from 2001 to 2010; and the network links in the left half of the network are mostly yellow, which means that these cocitations occurred most recently, between 2011 and 2020.

Additionally, to better understand the network structure and its content, it is important to identify special nodes and links. Special nodes take important positions in the knowledge network and play a specific role in the evolution of the knowledge structure; they can be identified flexibly through cocitation frequency, BC and half-life in CiteSpace. Highly cocited literature plays a fundamental role in the discipline (Table 4). A study with high centrality is an important turning point and milestone in the development of this research field (Table 5). The cited references represent the research basis, and the longer the half-life is, the more classic a study can be [31].

**Table 4:** Top 10 reference with the most co-citation of health literacy.

Rank	Counts	Author	Year	Reference	HalfLife	Cluster #
**1**	888	Berkman N. D.	2011	Low Health Literacy and Health Outcomes: An Updated Systematic Review	6	#0
**2**	746	Sorensen K.	2012	Health literacy and public health: A systematic review and integration of definitions and models	6	#3
**3**	268	Nutbeam D.	2008	The evolving concept of health literacy	6	#3
**4**	241	Sorensen K.	2015	Health literacy in Europe: comparative results of the European health literacy survey (HLS-EU)	4	#3
**5**	237	Jorm A. F.	2012	Mental health literacy: Empowering the community to take action for better mental health	6	#7
**6**	226	Osborne R. H.	2013	The grounded psychometric development and initial validation of the Health Literacy Questionnaire (HLQ)	5	#3
**7**	208	Schillinger D.	2002	Association of Health Literacy With Diabetes Outcomes	5	#1
**8**	202	Chew L. D.	2008	Validation of Screening Questions for Limited Health Literacy in a Large VA Outpatient Population	6	#0
**9**	200	DeWalt D. A.	2004	Literacy and Health Outcomes: Systematic Review Literacy and Health Outcomes	6	#1
**10**	194	Weiss B. D.	2005	Quick Assessment of Literacy in Primary Care: The Newest Vital Sign	6	#1

**Table 5:** Top 10 reference with the highest BC of health literacy.

Rank	BC	Author	Year	References	HalfLife	Cluster #
**1**	0.19	Kalichman S. C.	1999	Adherence to combination antiretroviral therapies in HIV patients of low health literacy	6	#2
**2**	0.18	Henderson S.	2000	Australia’s mental health: an overview of the general population survey	3	#4
**3**	0.18	Ware J. E.	1996	A 12item short-form health survey: Construction of scales and preliminary tests of reliability and validity	7	#5
**4**	0.14	Berkman N. D.	2011	Low Health Literacy and Health Outcomes: An Updated Systematic Review	6	#0
**5**	0.14	Clement S.	2015	What is the impact of mental health-related stigma on help-seeking? A systematic review of quantitative and qualitative studies	4	#7
**6**	0.13	Jorm A. F.	2012	Mental health literacy: Empowering the community to take action for better mental health	6	#7
**7**	0.11	DeWalt D. A.	2004	Literacy and Health Outcomes: Systematic Review Literacy and Health Outcomes	6	#1
**8**	0.10	WHO	2013	Health literacy: The solid facts	6	#3
**9**	0.09	Sorensen K.	2012	Health literacy and public health: A systematic review and integration of definitions and models	6	#3
**10**	0.09	Kutcher S.	2016	Mental Health Literacy: Past, Present, and Future	4	#7

First, Berkman’s *Low Health Literacy and Health Outcomes*: *An Updated Systematic Review* (n = 888), published in 2011, has been cocited most frequently. This paper also has high BC (BC = 0.14), showing that it not only has been cited many times but is also an important node connecting multiple studies. Based on a retrospective analysis of 111 English studies, this paper confirms that there is a correlation between low health literacy and poor health outcomes and poor use of health care services. Second, the study with the highest BC (BC = 0.19) is *Adherence to combination antiretroviral therapies in HIV patients of low health literacy*, published by Kalichman in 1999. The results of the intervention with HIV-seropositive patients showed that health literacy is an important independent predictor of treatment compliance. Generally, there was a burst of publication of these important node studies in 2005–2015.

#### 3.3.2. Analysis of cocitation clusters

In this paper, CiteSpace’s clustering function is used to classify the cocited studies and draw the knowledge map of cocitation clusters to objectively and scientifically summarize the research hot spots in the field of health literacy. A cluster of cited references forms the foundation of knowledge, while the citing articles form the research frontier [31]. The main advantage of this method is that it enables researchers to consider multiple aspects of citation relations from multiple perspectives. For the cluster map of health literacy research, modularity (Q value) and silhouette (S value) are two important indicators showing the characteristics of the overall structure of clustering networks. Modularity is an evaluation index that was proposed by E.J. Newman in 2004 [45], which depicts the quality of the community structure, and its formula is as follows:

$$Q=\sum_{i}(e_{ii}-a_{i}^{2})$$

where “i” is the number of the divided community, “e” presents the proportion of the links inside the community to all the links in the whole graph, and **a**_**i**_ is the proportion of the links associated with community i to all links. The better the clustering result is, the more internal links there will be, and thus, the greater the **e**_**i**_ value, the higher the Q value. Q values are generally in the interval [0,1], and Q > 0.3 (empirical data) is indicative of a true community structure. The silhouette value, proposed by Kaufman and Peter Rousseeuw in 1990 [46], is another parameter for evaluating clustering results, and the silhouette coefficient for a sample is s_i_ = 1 − a/b, where a is the average distance between node i and the other nodes in its cluster, and b is the average distance between the sample and the nearest cluster of which the sample is not a part. The average silhouette is the average of all samples. Clustering is efficient and convincing when the silhouette value is 0.7 and is generally considered reasonable when the value is above 0.5.

According to the output results, the Q value of the map is 0.6239, greater than 0.3, meaning that the module structure of clustering is significant, and the S value is 0.85, greater than 0.7, meaning that the clustering result is reasonable [31]. In the cluster, the lowest S value is 0.577 (#0), and the highest is 0.996 (#7), meaning that the homogeneity is relatively high [31].

**Fig 8:**
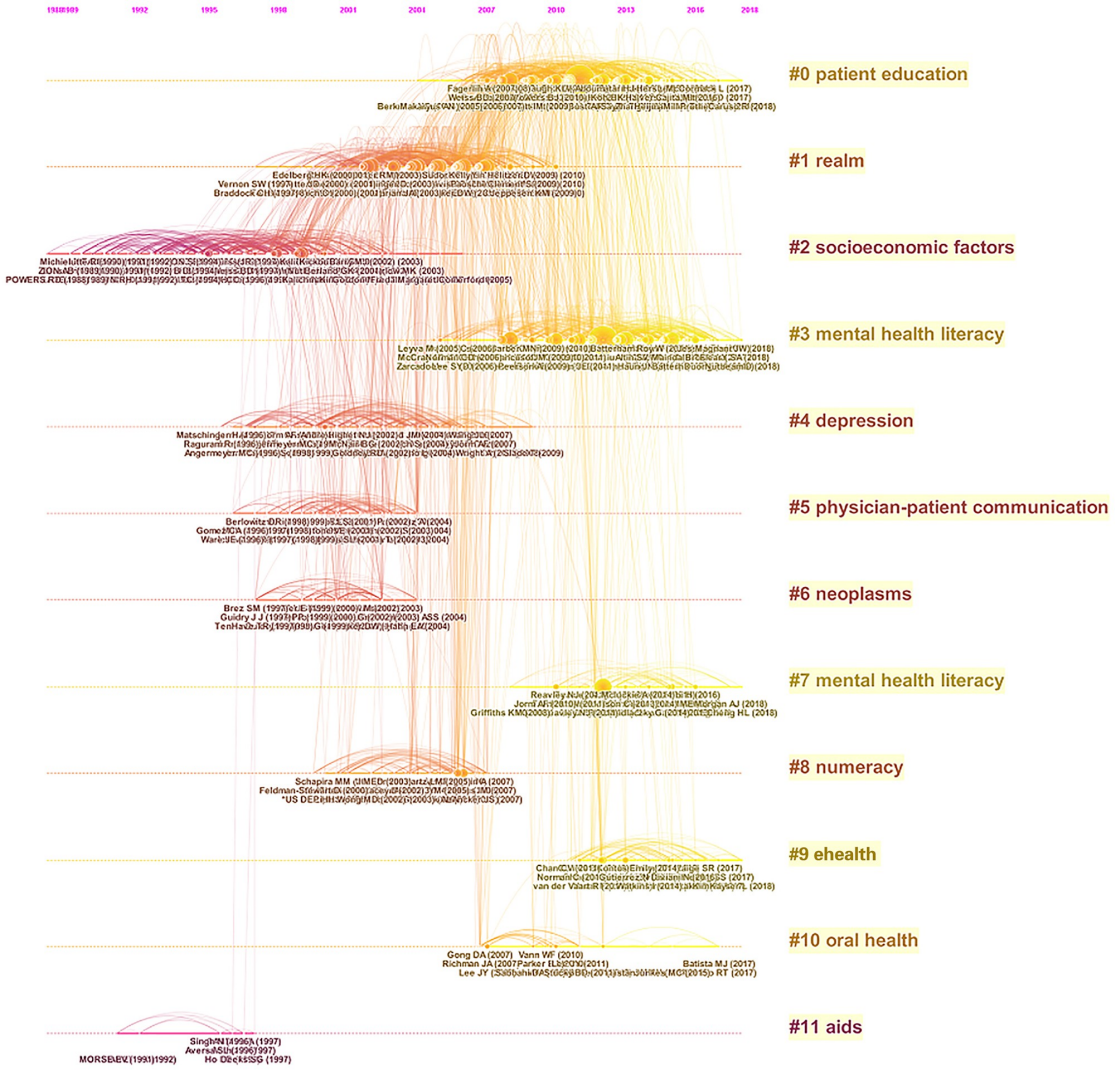
Timeline view of health literacy. This figure presents 12 clusters, which are arranged and numbered in ascending order from #0 to #11, with the colors corresponding to the average year in which the clusters were active. The larger the node, the more times it was cocited. Each cluster represents an area that has been developed or is developing Hence, the closer the arch body is to the right, the newer the topic.

To provide a more intuitive understanding of the evolution of research hot spots over time, a timeline view of the clustering results (Fig 8) sets each node in the position corresponding to the cluster to which the node belongs (axis of ordinates) and the publication time (abscissa axis) of the node. The nodes of the same cluster are arranged on the same level line in time order, displaying the historical results of the cluster. Compared with the relationship within the cluster, the timeline view pays more attention to the relationship among the clusters. In the timeline view, all 12 clusters are arranged from #0 to #11 in ascending order, with their color matching the years in which the clusters were active. Large nodes usually indicate a high level of referencing, a citation burst, or both.

The automatic clustering function and the clustering tags automatically extracted from the titles of citing articles based on the log-likelihood ratio vb algorithm can help us understand the content of the health literacy cocitation network. Each cluster represents a field that has been developed or is developing. The closer the para-curve is to the right, the newer the topic is. The graph also shows that the durations of different clusters are not consistent. Some topics, such as socioeconomic factors (#2) and realm (#1), have left footprints in the history of promoting research on and the development of health literacy, while mental health literacy (#7) and patient education (#0) are clusters that have been active in recent years. In addition, there are emerging themes such as electronic health (eHealth) (#9).

## 4. Discussion

### 4.1. Evolution of collaboration networks in health literacy research

In recent years, an increasing number of scholars have become aware of the importance of scientific collaboration, which has become an important way to promote the development of science and technology, economy and society as a means of scientific knowledge production. This study provides a panoramic view of the collaboration network and academic influence of health literacy research through four types of collaboration network analysis provided by CiteSpace: countries/regions, institutions, authors and interdisciplines. First, from the perspective of geographical distribution and the number of papers, the distribution of health literacy research papers is highly unbalanced. From a global perspective, the United States and Australia have the largest number of health literacy papers. The two countries have attached great importance to this topic, resulting in an increasing number of papers on a yearly basis. In addition, Canada, the Netherlands and other developed countries have contributed a great number of papers. Some Asian countries, such as China and Japan, have also stepped up their efforts in health literacy research, and the number of papers from these two countries is in the top 10 globally.

Second, the results of the BC measures of the global health literacy research collaboration network show that the United States, Australia and the United Kingdom are the major forces in the current international collaboration network on health literacy, occupying important positions in the global collaboration on health literacy research and playing important roles in linking cross-border collaboration on health literacy research. BC measures of research institutions show that most of the collaboration networks are based in European and American countries. Northwestern University in the United States is the most productive and influential research institution in the field of health literacy, with a great number of publications and high BC. Additionally, the University of Melbourne and University of Sydney in Australia are also key forces in international health literacy collaboration, with BC measures and number of papers ranking in the top five, indicating that they play an important linking role in promoting collaboration among countries. It is worth noting that although there is close collaboration among universities in the same country/region, there are still relatively very few transnational collaborations between Eastern and Western research institutions. From the perspective of collaboration among authors, Michael S. Wolf is the most influential author in the field of health literacy research; he has established a strong collaboration team, which has had a great impact on the overall structure of the collaboration network of authors worldwide.

Although the importance of geographical distance in collaboration has been weakened by the development of information and communication technologies, the distribution of collaborative relationships in health literacy research remains closely linked to geographical locations. Within the international network of health literacy, North America, with the United States at its core, Asian countries, with Japan and China as their main forces, and European countries are just like the three legs of a tripod. Although collaboration between transnational institutions has become more common, collaboration within national institutions remains the dominant trend, and the geographically closer the institutions are located, the more prominent the collaboration is. Additionally, collaboration among authors also exhibits some geographical proximity, with cooperation generally arising between researchers at the same institution. Thus, the collaboration networks of country/region, institution, and author present some variability.

Finally, the analysis of interdisciplinary collaboration in health literacy research shows that interdisciplinary collaboration in the field of health literacy was very rare at an earlier stage but began to flourish recently, involving more fields. Collaborations in public environmental occupational health, health care science services, nursing, general internal medicine and health policy services have been active in the past five years. This shows that research on health literacy has shifted from mere macrolevel research to microlevel research, from an independent discipline to interdisciplinary research. Thus, interdisciplinary collaboration should be a trend of future health literacy research.

### 4.2. Hot spots in health literacy research

This paper identifies the hot topics in the field of health literacy by integrating the classification of representative studies and cocitation clusters. To simplify the analysis, clusters are divided into active clusters and silent clusters, and information about certain important clusters is selected for discussion. Active and silent clusters are relative concepts, with the former meaning that the research has entered a new stage, representing emerging topics of research, and the cited references constitute the knowledge base of the research, while the key nodes are the literature that needs to be highlighted. Active clusters are restricted to clusters that have witnessed citation bursts in the most recent decade, while other clusters are silent, with burst that occurred before 2010 (Table 6).

**Table 6:** Major clusters of co-cited references of health literacy.

Cluster#	Size	Silhouette	Begin Year	End Year	Mean Year	Top Terms (log-likehood ratio, p-level)
**#0**	167	0.577	2004	2018	2011	patient education
**#1**	165	0.580	1997	2010	2005	realm
**#2**	154	0.821	1988	2006	1997	socioeconomic factors
**#3**	115	0.668	2005	2018	2012	mental health literacy
**#4**	92	0.983	1996	2009	2002	depression
**#5**	59	0.878	1996	2004	2000	physician-patient communication
**#6**	44	0.874	1997	2004	2001	neoplasms
**#7**	31	0.996	2008	2018	2013	mental health literacy
**#8**	27	0.950	2000	2007	2004	numeracy
**#9**	24	0.965	2011	2018	2014	ehealth
**#10**	17	0.983	2007	2017	2010	oral health
**#11**	9	0.993	1991	1997	1995	aids

First, from the change in time order, it can be seen that the socioeconomic factors (#2) and AIDS (#11) clusters are the two themes that take form earliest and thus can be considered the knowledge base of health literacy. The socioeconomic factors cluster (#2) has 154 items, spanning from 1988 to 2006, 18 consecutive years. The S value is 0.821, showing high homogeneity. According to the timeline view, although the scale of the cluster is relatively large, it is no longer active. The starting point of the timeline is *Emergency Department Patient Literacy and the Readability of Patient-Directed Materials*, published by Powers R. D. in 1988. According to the annual average value, cluster #2 occurred mainly in the 1990s, which is in the early stage of health literacy research and development. The cluster is closely related to the network structure of several emerging clusters, establishing an important knowledge base for health literacy research. Cluster #11 contains 9 members, which emerged mainly in 1997. The relationship of the network links shows that this cluster is independent of all other clusters. The references in cluster #11 are all from a citing article–*Barriers to HIV/AIDS Treatment and Treatment Adherence among African-American Adults with Disadvantaged Education*, published by S. C. Kalichman in 1995. Through an intervention study of African American adult AIDS patients, this paper shows that education and health literacy are important factors in adhering to HIV treatment and gaining access to medical services.

Clusters #1, #4, #5, #6 and #8 are in the central area of the horizontal axis of the timeline view and have a close relationship with cluster #2, indicating a major stage of the development of health literacy research. Cluster #1 spans from 1997 to 2010, 13 consecutive years, with 165 items and an S value of 0.58, indicating low homogeneity and uncertainty in the clustering tags. Although clusters #4, #5, #6 and #8 are relatively independent of other clusters, they represent objects and characteristics of different subsets of health literacy research. Different intermediary and explanatory mechanisms are adopted to prove that health literacy plays an important role in the treatment of depressive moods [47], establishing a good relationship between doctors and patients [48], and rehabilitation of cancer patients [49].

In the third stage of health literacy research (2005–2020), patient education (#0) is the largest cluster, containing 167 documents. Cluster #0 spans from 2004 to 2018, showing that it is a topic that has enjoyed enduring popularity. The S value of the cluster is 0.541, which is relatively low among all the clusters, indicating that there is a tendency for this cluster to generate new topics. In addition, the articles with the highest cocitations are all from cluster #0. Another active and large topic that spans a long time is mental health literacy. Two clusters, #3 and #7, formed as a result of automatic clustering, which shows two different tendencies of citations on mental health literacy. References in cluster #3 are cited mainly by papers on assessment methods and practices of mental health literacy [50, 51], while the citation relationship of cluster #7 shows that the mental health literacy of adolescents is an important research direction [52, 53].

The eHealth cluster (#9) is an active cluster that began to emerge in 2011 and has been an emerging theme in the most recent decade. There are 24 items in the cluster, and its S value is 0.965, showing that the internal homogeneity is very high. With the rapid development of information technology, information and communication technology have had a great impact on all aspects of social life, including medical and health care. Therefore, how to improve the capabilities of the public to make full use of eHealth resources in an information-centered environment has gradually attracted the attention of researchers, and eHealth literacy has become a new field of health literacy research that has developed rapidly in recent years. In 2005, the World Health Organization defined eHealth as “the dissemination of health resources and health care information through electronic means, so that health professionals and users can disseminate and access health information” [54]. Based on the concept of eHealth, Norman and Skinner (2006) [55] first proposed the concept of eHealth literacy, which refers to the ability to search for, identify, understand and evaluate health information from electronic resources and process and apply the acquired information to solve health problems. In recent years, research on electronic health literacy has gradually been extended to a variety of different subgroups, such as teenagers [56], the elderly [57], medical professionals [58], and HIV-positive patients [59].

### 4.3. Trends and frontiers in health literacy research

Through a cocitation network and clustering analysis of the literature, this part defines the development trends of health literacy research over time. First, health literacy research has focused on conceptual frameworks and operational methods since 1995. At the second stage, between 2000 and 2004, the focus shifted to the comprehensive discussion of social factors of health literacy, making great contributions to research design, intermediary mechanisms and explanatory mechanisms. At the third stage, from 2005 to 2020, empirical research and theory on health literacy began to diversify gradually. On the basis of an enriched research design, scholars have applied more theoretical frameworks to explain health literacy.

This paper uses the burst detection technology provided by CiteSpace to reveal the frontier literature in the field of health literacy. The concept of the research frontier was first introduced by Price in 1965. A cocitation burst can detect emerging trends and sudden shifts of attention in the scientific development of disciplines. The algorithm behind burst detection is derived mainly from Kleinberg’s (2002) [60] emergent measurement algorithm, using numerical values to express the strength of the burst. The greater the value is, the greater the strength of the burst and the more obvious the development trend of the topic related to the burst. The large amount of citation data has led to a large number of emergent studies. We selected references that experienced great breakthroughs in the past five years, which represent the latest research frontiers of health literacy. Table 7 shows the top ten references in terms of strength of burst in the most recent five years, including empirical studies by Sorensen K. (2015) [61], Batterham R. W. (2016) [62], and Kutcher S. (2016) [51]. We predict that these citation bursts will continue to receive attention in the future because they are usually predictors of future research trends.

**Table 7:** Recent burst references of health literacy (2015–2020).

Rank	Strength	Author	Year	References	Begin	End
1	160.11	Berkman N. D.	2011	Low Health Literacy and Health Outcomes: An Updated Systematic Review	2015	2020
2	158.09	Sorensen K.	2012	Health literacy and public health: A systematic review and integration of definitions and models	2016	2020
3	80.69	Sorensen K.	2015	Health literacy in Europe: comparative results of the European health literacy survey (HLS-EU)	2017	2020
4	54.13	Osborne R. H.	2013	The grounded psychometric development and initial validation of the Health Literacy Questionnaire (HLQ)	2016	2020
5	42.34	Jorm A. F.	2012	Mental Health Literacy: Empowering the Community to Take Action for Better Mental Health	2015	2020
6	36.35	Sorensen K.	2013	Measuring health literacy in populations: illuminating the design and development process of the European Health Literacy Survey Questionnaire (HLS-EU-Q)	2017	2020
7	33.87	Batterham R. W.	2016	Health literacy: applying current concepts to improve health services and reduce health inequalities	2017	2020
8	30.54	Berkman N. D.	2011	Health literacy interventions and outcomes: an updated systematic review	2015	2020
9	28.49	Gulliver A.	2010	Perceived barriers and facilitators to mental health help-seeking in young people: a systematic review	2015	2020
10	28.00	Kutcher S.	2016	Mental Health Literacy: Past, Present, and Future	2018	2020

## 5. Conclusions

This paper analyzes the network structure and trend of themes in health literacy research from 1995 to 2020 by using a multilayer collaboration network and multidimensional cocitation analysis. CiteSpace knowledge mapping analysis is panoramic. It can not only display the historical value of health literacy research but also show the main trends and future hot topics of health literacy research in recent years. The time distribution table of the literature shows that research on health literacy has been gradually expanding since the concept was first proposed. The main sources of health literacy literature are periodicals on medicine, public health and health-related fields, represented by *Patient Education and Counseling*, *Journal of General Internal Medicine* and *Journal of Health Communication*. The research on health literacy in the United States is way ahead of that in other countries, and its published literature accounts for more than 67% of the total literature. Moreover, interdisciplinary network analysis based on the map of scientific knowledge shows that research on health literacy is a process that shifts its focus from independent disciplinary research to interdisciplinary research. Finally, the key nodes in the literature and cluster classification reveal that research topics in health literacy research evolve over time: proposing and popularizing the concept of health literacy, introducing and improving evaluation tools, growing concerns for groups with low health literacy, generating a branch–mental health literacy, and putting forward emerging concepts such as eHealth literacy. In addition, mental health literacy, a relatively independent field in research and practice, has been the most active research field in recent years indicating that research in this field has matured over time. Sorensen K. (2015) [61], Batterham R. W. (2016) [62] and Kutcher S. (2016) [51] have provided frontier literature in the field of health literacy, which will be the main development trend of health literacy research and will also be the hot topics that will be the center of attention in the future.

Currently, knowledge about health literacy comes mainly through collaboration within a particular country or region, especially within an institution featured by a small group of 2–5 people. Moreover, the overall connectivity of co-authorship networks remains low, with subnetworks dominated by the dual-core and bridge pattern; international collaboration is mainly bilateral and trilateral, and the greatest level of international collaboration is found in developed areas such as Europe and the U.S. On a deeper level and in the long run, a systematic approach is needed to address health literacy issues, together with a strengthened framework of collaborative networks for health literacy at all regional, national and global levels. At the same time, more collaboration between research groups in different countries and institutions is encouraged to further drive the application of health literacy research forward. In the future, we anticipate that this research field will expand in two directions, namely, mental health literacy and eHealth literacy, both of which are closely linked to social development and issues.

First, mental health problems have become a key public concern, and one of the major causes of this phenomenon is the generally low mental health literacy of the population [63, 64]. An increase in mental health literacy can provide an optimized pathway for early interventions to avoid worsening psychological problems [65]. Therefore, research in this field deserves more attention from the academic and social sectors. Second, with the rapid development of online consultations, eHealth literacy has become an important competency indicator that directly influences how netizens access health information, use it and make relevant health choices. We should actively promote the measurement of eHealth literacy and conduct surveys among different population groups [66]. We should also use different interventions to improve their access as well as their ability to screen health information. In summary, this study can significantly complement traditional literature reviews and provide useful information for future directions and perspectives in health literacy research.

There are some deficiencies in this study. First, the sources of data are limited. Only six online databases are used, which may not cover all studies on health literacy. In addition, non-English publications are not included in our analysis, so publications in other languages should be considered in future research. Second, the strategy of selecting “health literacy” as the only search term can be improved. Specifically, future research should search for more keywords in a more flexible manner to find different research databases. Third, because the citation analysis focuses on the representative literature that has been cited, it is impossible to objectively evaluate newly published high-quality papers. Fourth, the technical analysis of our study relies on bibliographic records without considering the differences between the results of bibliometric analysis and the practical research conditions as well as the rich stories behind the relations of literature.

Additionally, the intrinsic defects of CiteSpace software will lead to inevitable errors in the process of data processing and transformation, such as unintegrated synonyms or ununified literature types. The data used by CiteSpace for analysis are quantitative, so in many cases, it is difficult to interpret the clusters directly from a qualitative perspective, whether it is a delineated collaborative network, an automatically generated cluster or a group of clustering labels obtained from the cocitations based on the TF*IDF weighting algorithm. This is surely a common problem faced by scholars when they adopt CiteSpace for visual analysis [21, 67–70]. Therefore, these descriptive labels usually require further manual classification, refinement and meaning.
